# Zinc Oxide and Graphitic Carbon Nitride/ZnO Nanocomposites in Food Safety and Preservation: Synthesis, Antimicrobial Mechanisms, and Application Prospects

**DOI:** 10.1002/fsn3.72509

**Published:** 2026-10-26

**Authors:** David Adeiza Zakari, Ibrahim Khalil, Kareem Sarafadeen Olateju, Sadullaev Tuymurod Mardullo ugli, Normirzaev Abdukayum Rakhimberdievich, Samandarov Abrorbek, Audu Godwin Amoka, Adefila Adebimpe Moyosore

**Affiliations:** ^1^ Department of Microbiology Prince Abubakar Audu University Anyigba Kogi State Nigeria; ^2^ Department of Microbiology, Faculty of Life Sciences Federal University Lokoja Lokoja Kogi State Nigeria; ^3^ Department of Microbiology, College of Biosciences Federal University of Agriculture Abeokuta Ogun State Nigeria; ^4^ Department of Energy and Applied Sciences Kimyo International University in Tashkent Tashkent Uzbekistan; ^5^ Candidate of Technical Sciences Namangan State Technical University (NamSTU) Namangan Uzbekistan; ^6^ Department of Food Technology Urgench State University Urgench Uzbekistan; ^7^ Construction Department, Institute of Technical Training University of Applied Science Engineering and Technology Banjul The Gambia

**Keywords:** antimicrobial packaging, food safety, graphitic carbon nitride, green synthesis, nanobiosensors, photocatalysis, S‐scheme heterojunction, zinc oxide nanoparticles

## Abstract

Foodborne disease remains a major global health and economic burden, with recent World Health Organization estimates attributing roughly 866 million illnesses and 1.52 million deaths annually to 42 major foodborne hazards. Conventional preservation and decontamination strategies are increasingly challenged by antimicrobial‐resistant pathogens, chemical residues, and mycotoxin contamination, creating demand for complementary nanotechnology‐based interventions. Zinc oxide (ZnO) nanoparticles and their heterojunctions with graphitic carbon nitride (g‐C_3_N_4_) have emerged as versatile platforms combining broad‐spectrum antimicrobial activity with visible‐light‐driven photocatalytic detoxification. This review synthesizes recent literature on the synthesis of ZnO nanoparticles by conventional and green/biogenic routes, including microbially mediated synthesis using actinobacteria such as *Streptomyces* species, and on the design of g‐C_3_N_4_/ZnO S‐scheme heterojunctions that suppress charge‐carrier recombination while preserving strong redox potentials. We examine the principal antimicrobial and photocatalytic mechanisms—reactive oxygen species generation, Zn^2+^ ion release, and direct membrane interaction—and their translation into food‐relevant applications, including antimicrobial active packaging films and coatings, photocatalytic degradation of mycotoxins and pesticide residues, and nanomaterial‐based biosensors for pathogen and spoilage detection. Toxicological and regulatory considerations, including nanoparticle migration from food‐contact materials and evolving European Food Safety Authority and United States Food and Drug Administration frameworks, are discussed alongside persistent gaps in standardized safety testing, scale‐up reproducibility, and life‐cycle assessment. We conclude by outlining research priorities—particularly the systematic evaluation of biogenic ZnO and g‐C_3_N_4_/ZnO composites under real food matrices and regulatory‐relevant conditions—needed to advance these materials from laboratory demonstration toward safe, standardized, and sustainable adoption across the food value chain.

## Graphical abstract

ZnO and g‐C_3_N_4_/ZnO S‐scheme heterojunctions are synthesized via conventional, green, and biogenic routes. They combat foodborne pathogens and degrade mycotoxins through ROS generation, Zn^2+^ release, and membrane disruption, enabling antimicrobial packaging, photocatalytic decontamination, and nanobiosensing. However, migration, toxicity, and regulatory validation gaps still limit real‐food adoption.

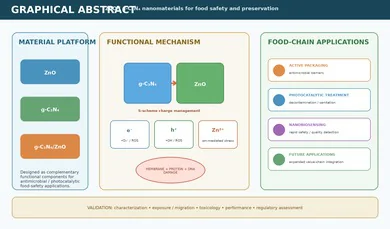

## 1 Introduction

Safe and adequate food supply remains one of the defining public health challenges of the twenty‐first century. The most recent World Health Organization/Foodborne Disease Burden Epidemiology Reference Group synthesis estimated that, in 2021, the 42 major foodborne infectious and chemical hazards under surveillance caused approximately 866 million illnesses, 1.52 million deaths, and 57.1 million disability‐adjusted life years globally, with the heaviest burden falling on children and populations in low‐ and middle‐income countries (WHO/FERG 2026). Diarrhoeal pathogens, mycotoxin‐contaminated staples, and chemical residues account for a large share of this burden, and the problem is compounded by the accelerating spread of antimicrobial‐resistant foodborne bacteria, which limits the effectiveness of conventional sanitizers and preservatives and has been projected to cause millions of additional deaths annually by mid‐century if left unaddressed.

Against this backdrop, nanotechnology has attracted sustained interest as a complementary strategy for food safety and preservation. Engineered nanomaterials—particularly metal oxide nanoparticles—offer antimicrobial activity through mechanisms that are mechanistically distinct from conventional antibiotics and biocides, which may reduce the risk of cross‐resistance while enabling multifunctional roles such as active packaging, contaminant degradation, and rapid pathogen sensing. Among metal oxide nanomaterials, zinc oxide (ZnO) nanoparticles occupy a prominent position because zinc is classified as Generally Recognized As Safe (GRAS) by regulatory agencies for specific bulk applications, ZnO is comparatively inexpensive to produce, and its wide direct band gap (~3.37 eV) confers both photocatalytic and antimicrobial functionality.

However, ZnO nanoparticles also present notable limitations that constrain their standalone utility. These include: (i) rapid electron–hole recombination that limits photocatalytic quantum efficiency; (ii) a wide band gap that restricts photoactivation primarily to ultraviolet (UV) light, which constitutes only a small fraction (~4%–5%) of ambient solar radiation and is largely absent in indoor food storage environments; (iii) potential Zn^2+^ leaching and associated toxicity concerns at elevated concentrations; and (iv) structural instability and photocorrosion under prolonged irradiation or in acidic and halide‐rich environments relevant to certain food matrices. These limitations have motivated the development of heterojunction architectures that extend functionality into the visible region while mitigating charge recombination.

More recently, coupling ZnO with graphitic carbon nitride (g‐C_3_N_4_), a metal‐free, visible‐light‐responsive polymeric semiconductor, has been explored to overcome the rapid electron–hole recombination and predominantly UV‐light activation that limit pristine ZnO, extending functional activity into the visible region of the solar spectrum that dominates ambient and indoor lighting conditions relevant to food storage and retail environments.

This review synthesizes recent literature (predominantly 2020–2026) on ZnO and g‐C_3_N_4_/ZnO nanocomposites as they pertain to food safety and preservation. The specific novelty and added value of this review, compared with recent reviews on ZnO nanomaterials in food safety, lie in its integrated treatment of ZnO and g‐C_3_N_4_/ZnO within a food‐safety framework; its explicit distinction between evidence obtained in model systems and evidence from real food matrices; its critical assessment of regulatory and toxicological considerations specific to food‐contact applications; and its identification of research priorities that bridge laboratory findings and industrial adoption.

We first summarize synthesis routes, contrasting conventional physicochemical methods with green and microbially mediated (biogenic) synthesis, an area of particular relevance given the soil‐microbial biosynthetic capacity increasingly documented for actinobacteria such as *Streptomyces* species. We then describe the design principles underlying g‐C_3_N_4_/ZnO S‐scheme heterojunctions and the shared mechanistic basis of their antimicrobial and photocatalytic activity. The review subsequently surveys three converging application domains: antimicrobial active packaging, photocatalytic decontamination of mycotoxins and pesticide residues, and nanomaterial‐based biosensing for pathogen and spoilage detection. Because uptake of these materials in the food sector depends as much on demonstrated safety as on efficacy, we dedicate a section to toxicological and regulatory considerations, including nanoparticle migration from food‐contact materials and the evolving positions of the European Food Safety Authority (EFSA) and the United States Food and Drug Administration (FDA). We close by identifying research gaps and priorities that, in our assessment, must be addressed before these nanomaterials can move from laboratory demonstration toward standardized, regulator‐endorsed use across the food value chain.

### 1.1 Literature Search Strategy

This review was compiled through systematic searches of PubMed, Scopus, Web of Science, and Google Scholar using combinations of the following search terms: “ZnO nanoparticles,” “zinc oxide,” “g‐C_3_N_4_,” “graphitic carbon nitride,” “food safety,” “antimicrobial packaging,” “photocatalysis,” “mycotoxin degradation,” “foodborne pathogens,” “green synthesis,” “biogenic synthesis,” “S‐scheme heterojunction,” and “nanobiosensors.” The primary inclusion criteria were: peer‐reviewed articles published in English; studies reporting original experimental data on synthesis, characterization, antimicrobial activity, photocatalytic performance, or toxicological assessment; and studies with direct or indirect relevance to food safety and preservation. The primary exclusion criteria were: non‐peer‐reviewed literature, conference abstracts without full data, and studies focused exclusively on environmental remediation without any food‐relevant component. The search period covered 2015–2026, with emphasis on literature published between 2020 and 2026. Primary research articles were prioritized for citation; where specific numerical data were reported, every effort was made to cite the original experimental study rather than a secondary review, and where the primary source could not be accessed, the “as cited in” convention was used to maintain transparency.

## 2 Zinc Oxide Nanoparticles: Synthesis and Properties

### 2.1 Conventional Physicochemical Synthesis

ZnO nanoparticles have historically been produced by a range of physical and chemical routes, including sol–gel processing, hydrothermal and solvothermal synthesis, mechanochemical processing, physical vapor synthesis, precipitation, and, more recently, microfluidic and laser‐ablation methods. These routes generally afford good control over particle size, morphology (spherical, rod, flower‐like, and tetrapod architectures have all been reported), and crystallinity, and they remain the dominant methods used in commercial‐scale production (Zhou et al. 2023; Gökmen et al. 2024). However, many conventional syntheses rely on organic solvents, surfactants, or elevated temperatures and pressures, raising concerns about cost, energy intensity, and the environmental and residual‐toxicity burden of the resulting nanomaterials when they are intended for direct contact with food (Gökmen et al. 2024). Furthermore, residual organic solvents or unreacted precursors may persist on nanoparticle surfaces, necessitating rigorous purification before food‐contact applications.

### 2.2 Green and Biogenic Synthesis

Green synthesis approaches use plant extracts, fungi, algae, or bacteria as reducing and capping agents, replacing hazardous chemical reductants with biological metabolites and thereby aligning nanoparticle production with green chemistry principles. Reviews of this literature note that, despite substantial work on plant‐mediated ZnO synthesis for antimicrobial and biomedical purposes, direct application of green‐synthesized ZnO nanoparticles within food packaging or food‐contact materials has been comparatively underexplored, representing a recognized gap between green‐synthesis research and food‐sector translation (Vieira et al. 2026).

Microbially mediated (biogenic) synthesis using bacteria, and actinobacteria in particular, has emerged as a scalable variant of green synthesis. Extracellular biosynthesis—in which the cell‐free supernatant of a microbial culture, rather than intact cells, supplies the nucleation‐directing and capping biomolecules—offers practical advantages including simplified downstream processing, reduced contamination from intracellular debris, and easier scale‐up. The mechanism generally involves: (i) complexation of Zn^2+^ ions by extracellular biomolecules (proteins, polysaccharides, enzymes); (ii) nucleation and growth of ZnO crystallites under the directing influence of these biomolecules; and (iii) capping and stabilization of the resulting nanoparticles by residual organic molecules.

Several *Streptomyces* species have been used to biosynthesize ZnO nanoparticles with confirmed antibacterial activity. ZnO nanoparticles synthesized using an estuarine‐associated *Streptomyces* sp. showed pronounced growth inhibition against both bacterial and fungal pathogens, with the strongest activity recorded against *Escherichia coli* (22 mm inhibition zone, particle size 51–65 nm; cited in review literature on microbial ZnO synthesis). ZnO nanoparticles synthesized using *Streptomyces plicatus* MK‐104 displayed activity against several plant‐pathogenic fungi and a nematicidal effect, illustrating dual food‐safety and agricultural relevance (particle size ~22 nm, MIC 15.6–500 μg/mL). ZnO nanoparticles synthesized with *Streptomyces baarnensis* MH‐133, combined with an actinobacterially derived bioactive metabolite in nanoemulsion form, showed enhanced activity against multidrug‐resistant ESKAPE pathogens together with favorable cytotoxicity profiles (particle size 12–17.5 nm; Kalaba et al. 2024). Most recently, ZnO nanoparticles biosynthesized from the cell‐free supernatant of an endophytic *Streptomyces werraensis* strain isolated from the medicinal plant *Passiflora caerulea* produced predominantly spherical, wurtzite‐phase nanoparticles with concentration‐dependent antibacterial activity against Gram‐negative pathogens ( *Salmonella paratyphi* A and *Proteus vulgaris* ) comparable to ampicillin, alongside notable radical‐scavenging (antioxidant) activity (particle size ~136 nm by TEM; inhibition zones against *S. paratyphi* A = 19.0 mm and *P. vulgaris* = 16.4 mm; ABTS IC_50_ = 22.15 μg/mL; Patel et al. 2026). A comparative overview of conventional, plant‐mediated, and biogenic (actinobacterial) synthesis routes, including representative systems, particle sizes, morphologies, advantages, limitations, and food‐related relevance, is provided in Table 1.

**TABLE 1:** Representative synthesis routes for ZnO nanoparticles relevant to food‐safety applications, contrasting conventional, plant‐mediated, and biogenic (actinobacterial) approaches.

Synthesis route	Representative system	Particle size	Morphology	Synthesis conditions	Reported outcome	Advantages	Limitations	Direct food‐related relevance	References
Chemical/physical	Sol–gel, hydrothermal, mechanochemical, vapor synthesis	20–75 nm	Spherical, rod, flower‐like, tetrapod	Organic solvents, high temperature/pressure	Good control of size/morphology; dominant industrial route	Precise control; scalable; reproducible	Solvent‐ and energy‐intensive; residual chemicals	Indirect (requires purification for food contact)	Zhou et al. (2023); Gökmen et al. (2024)
Plant‐mediated green synthesis	*Cassia fistula* fruit extract; babassu coconut mesocarp extract	~20–60 nm	Spherical	Aqueous extract, moderate temperature	ZnO NPs incorporated into chitosan/gelatin and biopolymer films with antibacterial activity against *E. coli* and *S. aureus*	Eco‐friendly; low cost; biocompatible	Batch variability; limited size control	Direct (packaging films tested on food)	Mirres et al. (2024)
Bacterial/actinobacterial (biogenic) synthesis	Estuarine *Streptomyces* sp.	51–65 nm	Spherical	Cell‐free supernatant, ambient conditions	Strongest inhibition against *E. coli* (22 mm zone)	Scalable; extracellular; low toxicity	Batch variability; residual biomolecules	Indirect (antimicrobial activity against foodborne pathogen)	Cited in review literature on microbial ZnO synthesis
Biogenic synthesis	*Streptomyces plicatus* MK‐104	~22 nm	Not specified	Cell‐free supernatant	Antifungal/nematicidal activity; MIC 15.6–500 μg/mL	Dual food‐safety and agricultural relevance	Limited food‐matrix validation	Indirect (plant‐pathogen and nematicidal)	
Biogenic synthesis	*Streptomyces baarnensis* MH‐133 + active metabolite	12–17.5 nm	Not specified	Cell‐free supernatant + nanoemulsion	Activity against multidrug‐resistant ESKAPE pathogens	Enhanced activity via combination	Limited food‐safety validation	Indirect (biomedical context)	Kalaba et al. (2024)
Endophytic biogenic synthesis	*Streptomyces werraensis* (endophyte of *Passiflora caerulea* )	~136 nm (TEM)	Spherical, wurtzite	Cell‐free supernatant	Inhibition zones against *S. paratyphi* A (19.0 mm) and *P. vulgaris* (16.4 mm) comparable to ampicillin; ABTS IC_50_ 22.15 μg/mL	Antioxidant + antibacterial; endophytic source	Large particle size; limited food‐matrix testing	Indirect (foodborne pathogens tested in vitro)	Patel et al. (2026)

Beyond actinobacteria, fungal‐mediated synthesis (e.g., using *Aspergillus*, *Fusarium*, and *Trichoderma* species) has been explored, with fungi offering advantages of high biomass production and secretion of diverse metabolites. Algal‐mediated synthesis (using both microalgae and macroalgae) has also been reported, leveraging the reducing and capping capacity of algal polysaccharides and polyphenols. Plant‐mediated synthesis remains the most extensively studied green route, utilizing extracts from leaves, fruits, seeds, and other tissues. Each biological system offers distinct advantages and limitations in terms of synthesis rate, particle size control, morphology, and scalability.

#### 2.2.1 Influence of Synthesis Conditions on Nanoparticle Properties

The properties of biogenic ZnO nanoparticles are strongly influenced by synthesis parameters including (i) precursor concentration (affecting nucleation rate and final particle size); (ii) pH (influencing Zn^2+^ hydrolysis, crystallite formation, and surface charge); (iii) temperature (affecting crystallinity, phase purity, and reaction kinetics); (iv) incubation time (affecting particle growth and aggregation); (v) calcination temperature (affecting crystallinity, surface defects, and oxygen vacancy concentration); and (vi) the nature and concentration of capping biomolecules (affecting surface chemistry, colloidal stability, and antimicrobial activity). The biological variability inherent in the use of microbial or plant extracts can translate into batch‐to‐batch differences in particle size distribution and polydispersity that are less pronounced in tightly controlled chemical synthesis routes.

#### 2.2.2 Food‐Safety Relevance and Residual Biomolecules

For food‐contact applications, an important consideration is the possible presence of residual biomolecules, metabolites, or other synthesis‐related components on biogenic ZnO surfaces. While these organic residues may contribute to biocompatibility and colloidal stability, their presence raises questions about potential allergenicity, immunogenicity, and long‐term stability under food storage conditions. Systematic characterization of residual organic content and its fate during food‐contact exposure remains an evidence gap requiring attention.

Collectively, these reports position soil‐ and plant‐associated actinobacteria, as well as other biological systems, as versatile, low‐toxicity “bio‐factories” for ZnO nanoparticle production, with direct relevance to the antimicrobial‐packaging and pathogen‐control applications discussed in Section 5.

Beyond bacteria, functionalization strategies—including surface modification of ZnO with organic ligands (carboxylic acids, silanes) and inorganic overlayers, and doping with transition or rare‐earth metals—have been used to tune band gap, surface charge, and antimicrobial potency, broadening the design space available for food‐relevant formulations (Alameen et al. 2024a, 2024b).

## 3 Graphitic Carbon Nitride and G‐C_3_N_4_/ZnO Heterojunctions

Graphitic carbon nitride (g‐C_3_N_4_) is a metal‐free, two‐dimensional polymeric semiconductor built from tri‐s‐triazine (heptazine) units, first drawing sustained research interest for its low cost, ease of bulk synthesis by thermal polymerization of nitrogen‐rich precursors (melamine, urea, thiourea, dicyandiamide), high thermal and chemical stability, and—crucially—its visible‐light responsiveness, a property that pristine ZnO lacks because of its wide band gap (Huang et al. 2025). These properties have made g‐C_3_N_4_ a benchmark visible‐light photocatalyst for environmental remediation, including the degradation of dyes, antibiotics, and pesticides in water (reviewed in Huang et al. 2025).

Pristine g‐C_3_N_4_, however, suffers from several limitations that constrain its standalone photocatalytic and antimicrobial efficiency: (i) a relatively high rate of photogenerated electron–hole recombination, which reduces quantum efficiency; (ii) a modest specific surface area (typically 10–50 m^2^/g for bulk material), limiting adsorption and active‐site density; (iii) limited visible‐light absorption efficiency beyond ~460 nm; (iv) poor dispersibility in aqueous media; and (v) difficulty in achieving intimate interfacial contact with other semiconductors. Coupling g‐C_3_N_4_ with ZnO to form a heterojunction addresses these limitations: because the conduction and valence band edges of the two semiconductors are offset, photogenerated charge carriers can be spatially separated across the interface, suppressing recombination while retaining strongly reducing electrons and strongly oxidizing holes on each respective semiconductor.

### 3.1 S‐Scheme Mechanism

Several recent studies describe the ZnO/g‐C_3_N_4_ couple as forming a step‐scheme (S‐scheme) heterojunction. In this configuration, upon contact between ZnO and g‐C_3_N_4_, electrons flow from the higher Fermi level to the lower Fermi level until equilibrium is reached, creating an interfacial electric field and band bending at the interface. Under irradiation, the weaker photogenerated electrons in the conduction band of ZnO recombine selectively with the weaker holes in the valence band of g‐C_3_N_4_ through the interfacial pathway, leaving the strongly reducing electrons in the g‐C_3_N_4_ conduction band and the strongly oxidizing holes in the ZnO valence band available to drive redox chemistry (Prathibha et al. 2025; Hassan et al. 2024).

### 3.2 Comparison of Heterojunction Types

It is instructive to contrast the S‐scheme with other heterojunction architectures. In a conventional type‐II heterojunction, both photogenerated electrons and holes are transferred to the component with lower conduction band and higher valence band potentials, respectively. While this achieves spatial separation, it sacrifices redox power because the carriers accumulate at the less reducing/lower oxidizing band edges. In a conventional Z‐scheme, a shuttle mediator (e.g., IO_3_^−^/I^−^, Fe^3+^/Fe^2+^) or solid‐state mediator facilitates recombination of weak carriers, but the mediator can introduce side reactions and stability issues. The S‐scheme achieves spatial separation while retaining maximum redox potential through selective recombination of weak carriers at the interface without requiring a diffusing mediator.

### 3.3 Evidence for S‐Scheme Mechanism

It is important to emphasize that the S‐scheme designation should not be applied universally to all g‐C_3_N_4_/ZnO composites; the actual charge‐transfer mechanism is material‐ and interface‐dependent and must be verified experimentally for each system. Photocatalytic performance or radical‐scavenging experiments alone cannot definitively distinguish S‐scheme from type‐II mechanisms. Supporting evidence may include: in situ X‐ray photoelectron spectroscopy (XPS) under irradiation (showing binding energy shifts indicative of charge transfer direction); Kelvin probe measurements (work function determination and Fermi‐level equilibration); Mott–Schottky analysis (flat band potential and carrier density); electron paramagnetic resonance (EPR) spectroscopy (radical identification and quantification); and density functional theory (DFT) calculations (band structure and interfacial charge distribution). Where such evidence is available, it should be discussed; where it is absent, the mechanism should be described as “proposed” or “consistent with” an S‐scheme rather than definitively assigned.

Experimental support for this design comes from several directions. A solar‐matched ZnO/g‐C_3_N_4_ S‐scheme composite achieved substantially higher visible‐light degradation efficiency toward the pharmaceutical contaminant paracetamol than either pristine component, with an optimized loading (10 wt% g‐C_3_N_4_) more than doubling the reaction rate constant relative to lower‐loading composites (Hassan et al. 2024). Density‐functional‐theory calculations combined with radical‐scavenging experiments have likewise confirmed S‐scheme charge transfer and identified superoxide and hydroxyl radicals as the dominant reactive species responsible for accelerated dye degradation in g‐C_3_N_4_/ZnO composites relative to the individual semiconductors (Prathibha et al. 2025).

### 3.4 Modification Strategies

Extension of this design to ternary and doped systems has further improved degradation kinetics and antibacterial/disinfection performance under visible light. N,O dual‐vacancy Ce‐ZnO/g‐C_3_N_4_ systems introduce mid‐gap states that extend visible‐light absorption and serve as adsorption sites; Ce^3+^/Ce^4+^ redox couples facilitate charge transfer; Ag/ZnO/g‐C_3_N_4_ nanofiber heterojunctions create Schottky barriers that promote electron extraction and can contribute plasmonic enhancement. These modifications illustrate a broader strategy of defect and dopant engineering layered onto the base S‐scheme architecture (Figure 1).

**FIGURE 1:**
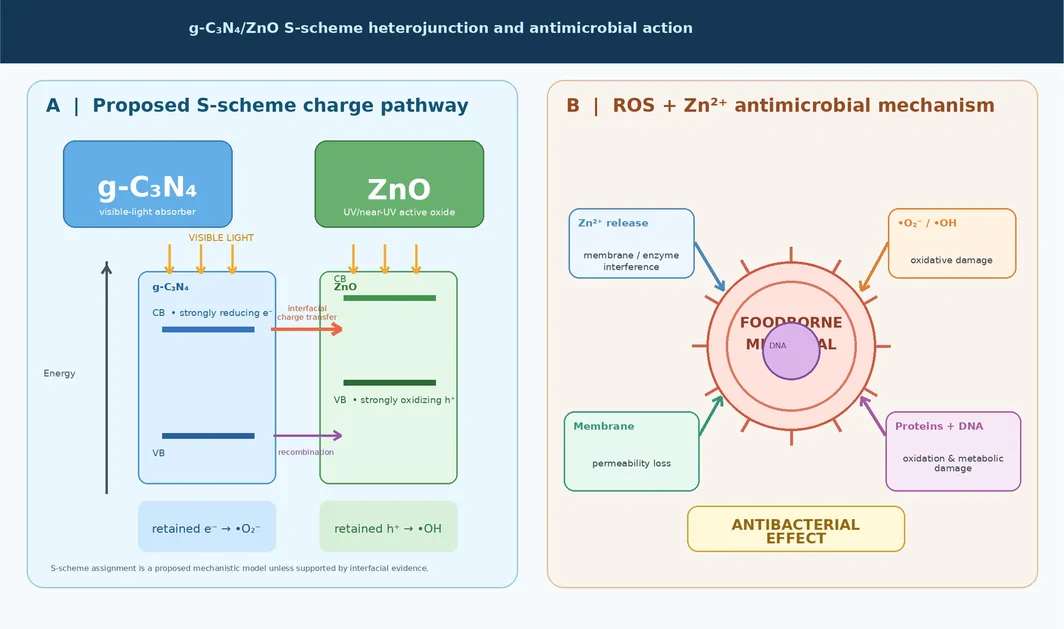
(A) Schematic S‐scheme band alignment and interfacial charge‐transfer pathway for a g‐C_3_N_4_/ZnO heterojunction under visible‐light irradiation, showing retention of strongly reducing electrons in g‐C_3_N_4_ and strongly oxidizing holes in ZnO. (B) Proposed reactive‐oxygen‐species‐mediated antibacterial mechanism, combining Zn^2+^ ion release, membrane disruption, and oxidative damage to proteins and DNA.

## 4 Antimicrobial and Photocatalytic Mechanisms

### 4.1 Photocatalytic Mechanisms

The antimicrobial and photocatalytic activities of ZnO and g‐C_3_N_4_/ZnO nanomaterials share a common mechanistic root. Under illumination with energy equal to or exceeding the semiconductor band gap, electrons are excited from the valence band to the conduction band, generating electron–hole pairs. In an S‐scheme g‐C_3_N_4_/ZnO heterojunction, interfacial recombination of the weaker carrier pair leaves highly reducing electrons concentrated in g‐C_3_N_4_ and highly oxidizing holes concentrated in ZnO. These charge carriers subsequently react with adsorbed water, dissolved oxygen, or hydroxide ions at the nanoparticle surface to generate reactive oxygen species (ROS), principally superoxide radical anions (˙O_2_^−^), hydroxyl radicals (˙OH), and hydrogen peroxide (H_2_O_2_) (Huang et al. 2025).

The major reactions responsible for ROS formation are:

- e^−^ + O_2_ → ˙O_2_^−^
- ˙O_2_^−^ + H^+^ → HO_2_˙
- 2HO_2_˙ → H_2_O_2_ + O_2_
- h^+^ + H_2_O → ˙OH + H^+^
- h^+^ + OH^−^ → ˙OH

For photocatalytic detoxification applications, the same ROS pool that damages microbial cells also attacks the double bonds, aromatic rings, and heteroatom‐containing functional groups of mycotoxins, pesticide residues, and synthetic dyes, progressively fragmenting them into smaller intermediates and, under favorable conditions, mineralizing them to carbon dioxide and water (Huang et al. 2025). However, it is critical to distinguish between parent compound disappearance (degradation), complete mineralization to CO_2_ and H_2_O, and actual toxicity reduction. Partial degradation can generate transformation products that may retain or even exhibit increased toxicity, and degradation percentage alone does not demonstrate detoxification. Toxicity assessment of degradation products, ideally using bioassays, is necessary to establish detoxification.

### 4.2 Antimicrobial Mechanisms

For antimicrobial action specifically, three interacting mechanisms are typically invoked, and their relative contributions depend on nanoparticle characteristics, concentration, target organism, and environmental conditions:

- ROS‐mediated oxidative damage to bacterial membrane lipids, proteins, and nucleic acids;
- Direct physical interaction between nanoparticles and the cell envelope, causing membrane disruption and increased permeability; and
- Dissolution and release of Zn^2+^ ions, which interfere with membrane transport, respiratory enzymes, and metabolic function (Patel et al. 2026; Alameen et al. 2024b).

It is important to distinguish light‐dependent mechanisms (ROS generation, which requires photoactivation) from dark mechanisms (Zn^2+^ release, direct nanoparticle‐cell contact, which operate under both illuminated and dark conditions). This distinction is critical for understanding performance in food storage environments, which may include dark conditions.

Because the mechanism does not depend on a specific molecular target in the way that many conventional antibiotics do, ZnO‐ and g‐C_3_N_4_/ZnO‐based antimicrobials are considered less susceptible to the single‐point‐mutation resistance mechanisms (e.g., β‐lactamase production) that underlie much conventional antimicrobial resistance. However, this statement should be presented cautiously: direct evidence from foodborne microorganisms is limited, and the potential for cross‐adaptation and sub‐lethal‐dose selection effects warrants continued monitoring (Patel et al. 2026).

#### 4.2.1 Distinguishing the Roles of ZnO, g‐C_3_N_4_, and the Heterojunction

ZnO contributes Zn^2+^ release, UV‐activated ROS generation, and direct contact‐mediated membrane disruption. g‐C_3_N_4_ contributes visible‐light‐activated ROS generation and adsorption capacity. The g‐C_3_N_4_/ZnO heterojunction creates emergent functionality through interfacial charge transfer: enhanced charge separation, extended visible‐light activity, sustained Zn^2+^ release, and synergistic membrane disruption. The heterojunction does not simply combine the properties of its components but enables new mechanistic pathways.

#### 4.2.2 Gram‐Negative vs. Gram‐Positive Susceptibility

Susceptibility differences between Gram‐negative and Gram‐positive bacteria are frequently reported, with Gram‐negative organisms often showing greater sensitivity in many studies. This has been attributed to their thinner peptidoglycan layer and lipopolysaccharide‐rich outer membrane, which is thought to facilitate electrostatic attraction of positively charged nanoparticle surfaces and subsequent penetration; the thicker peptidoglycan layer of Gram‐positive bacteria, by contrast, appears to impede nanoparticle access and slow ROS‐mediated damage (Patel et al. 2026). However, this pattern is not universal and depends strongly on nanoparticle characteristics (size, surface charge, morphology), concentration, and experimental conditions. Some Gram‐positive foodborne pathogens, such as *Listeria monocytogenes* , exhibit substantial resistance due to their thicker peptidoglycan layer and modified surface properties. Additional important foodborne pathogens including *Listeria monocytogenes* , *Staphylococcus aureus* , and *Bacillus cereus* should be considered in future studies to provide a broader assessment of antimicrobial spectrum.

#### 4.2.3 ROS Scavenging by Food Components

An important consideration for food applications is the possible scavenging of ROS by proteins, lipids, antioxidants, and other food components. Proteins and amino acids (particularly cysteine and methionine) can quench ROS; lipids can undergo peroxidation, consuming ROS and generating secondary products; antioxidants (e.g., ascorbic acid, polyphenols) can compete with target contaminants. The net effect is matrix‐dependent and difficult to predict without experimental validation.

## 5 Applications in Food Safety and Preservation

### 5.1 Antimicrobial Active Packaging and Coatings

The best‐developed food application of ZnO nanoparticles is their incorporation into active packaging films and edible or surface coatings, where they confer antimicrobial and, in some formulations, antioxidant functionality while often improving mechanical and barrier properties of the base polymer. Chitosan/gelatin hybrid films reinforced with green‐synthesized ZnO nanoparticles (derived from *Cassia fistula* fruit extract) showed improved thermal stability, elongation‐at‐break, and antimicrobial activity against both *E. coli* and *S. aureus* . Similarly, nanocomposite films combining babassu coconut mesocarp with green‐synthesized ZnO nanoparticles reduced *Staphylococcus aureus* contamination on cooked turkey ham samples in direct food‐contact trials, illustrating translation from polymer characterization toward real food matrices (Mirres et al. 2024). Alginate‐based composite films incorporating zinc‐based inorganic antimicrobials have likewise been shown to modulate antibacterial performance according to particle morphology, underscoring that synthesis route and morphology choices carry through to functional packaging performance (Zhang et al. 2024).

For packaging studies, key parameters including film thickness, nanoparticle loading, mechanical properties, water‐vapor/oxygen permeability, antimicrobial performance, and migration behavior should be reported where available to enable meaningful comparison across studies. The potential release of ZnO nanoparticles and Zn^2+^ ions from food‐packaging materials depends on factors including polymer type, nanoparticle loading, food simulant composition, temperature, and contact time, and requires systematic migration testing under standardized conditions.

A recent comprehensive review appearing in this journal traced the trajectory of ZnO nanomaterials in food‐related contexts from early solvothermal and sol–gel preparations toward more sustainable synthesis and risk‐assessment‐oriented framing, underscoring both the breadth of demonstrated antimicrobial efficacy against foodborne pathogens and the parallel need to reconcile that efficacy with safety and sustainability considerations (Gökmen et al. 2024).

### 5.2 Photocatalytic Decontamination of Mycotoxins and Pesticide Residues

Beyond packaging, the photocatalytic properties of ZnO and g‐C_3_N_4_‐based materials have been explored as a green, low‐cost route for detoxifying mycotoxin‐ and pesticide‐contaminated food and feed matrices. The Food and Agriculture Organization has previously indicated that over one‐quarter of global cereal production may be affected by mycotoxin contamination in a given year, and photocatalytic advanced oxidation is increasingly investigated as a complement to physical sorting and thermal treatment for reducing this burden (as summarized in Huang et al. 2025).

g‐C_3_N_4_‐based photocatalysts have shown strong activity against aflatoxin B1 (AFB1): flexible electrospun PCL‐g‐C_3_N_4_/carbon‐quantum‐dot membranes achieved degradation exceeding 95% for AFB1 solutions within 30 min of visible‐light exposure through combined adsorption and photocatalysis (as reviewed in Huang et al. 2025), while g‐C_3_N_4_ catalysts derived from different nitrogen‐rich precursors (dicyandiamide, melamine, thiourea, urea) have been systematically compared for AFB1 degradation efficiency under visible light. ZnO/graphene hybrids have similarly achieved near‐complete degradation of deoxynivalenol under ultraviolet irradiation at rates several‐fold higher than pristine ZnO (as reviewed in Huang et al. 2025), and g‐C_3_N_4_ synthesized by conventional thermal polymerization has degraded zearalenone under ultraviolet exposure, with somewhat lower efficiency observed for zearalenone embedded in complex real food‐powder matrices than for reference standards in solution—a discrepancy that recurs across this literature and is discussed further in section 7 (Huang et al. 2025). Representative photocatalytic degradation performance data for g‐C_3_N_4_‐ and ZnO‐based systems against food‐relevant mycotoxins, dyes, and model organic contaminants—including light source, irradiation time, contaminant concentration, degradation efficiency, system type, reusability, and toxicity assessment—are summarized in Table 2.

**TABLE 2:** Representative photocatalytic degradation performance of g‐C_3_N_4_‐ and ZnO‐based systems against food‐relevant mycotoxins, dyes, and model organic contaminants.

Target contaminant	Photocatalyst system	Light source	Irradiation time	Contaminant concentration	Degradation efficiency	System type	Reusability	Toxicity assessment	References
Aflatoxin B1 (AFB1)	PCL‐g‐C_3_N_4_/carbon quantum dot electrospun membrane	Visible light	30 min	Not specified	> 95%	Model solution	Membrane reusable/regenerable	Not reported	Reviewed in Huang et al. (2025)
Aflatoxin B1 (AFB1)	g‐C_3_N_4_ from varied N‐rich precursors	Visible light	Not specified	Not specified	Precursor‐dependent	Model solution	Not reported	Not reported	
Deoxynivalenol (DON)	ZnO/graphene hybrid	UV	30 min	15 ppm	~99%	Model solution	Not reported	Not reported	Reviewed in Huang et al. (2025)
Zearalenone (ZEN)	g‐C_3_N_4_ (thermal polymerization)	UV (254 nm)	Not specified	Not specified	96.0% (reference); 50.0% (real powder matrix)	Model solution and real food powder	Not reported	Not reported	Reviewed in Huang et al. (2025)
Azo dye + *E. coli* (fruit juice)	Porous g‐C_3_N_4_ tubes with biomass‐derived carbon dots	Visible light	Not specified	Not specified	Simultaneous dye reduction and bacterial inactivation	Fruit juice (real matrix)	Not reported	Not reported	
Paracetamol (model pharmaceutical contaminant)	ZnO/g‐C_3_N_4_ S‐scheme heterojunction (10 wt% g‐C_3_N_4_)	Visible light	60 min	Not specified	95%	Model solution	Not reported	Not reported	Hassan et al. (2024)

*Note:* Paracetamol is included as a model pharmaceutical contaminant to illustrate S‐scheme photocatalytic performance; it is not a representative food contaminant.

For pesticide and other organic residues, g‐C_3_N_4_ and its heterojunctions have been used extensively for wastewater remediation, degrading dyes, antibiotics, and pesticide residues under visible light through the same ROS‐driven advanced‐oxidation pathway described in Section 4. This remediation chemistry is increasingly being repurposed for direct food‐matrix and process‐water decontamination, including photocatalytic reduction of an azo dye and simultaneous inactivation of *Escherichia coli* in fruit juice using porous g‐C_3_N_4_ tubes anchored with waste‐biomass‐derived carbon dots. Ternary and doped heterojunctions, such as Bi_7_O_9_I_3_/g‐C_3_N_4_/ZnO and Ag/ZnO/g‐C_3_N_4_ nanofiber composites, extend this dual disinfection‐and‐degradation functionality, achieving simultaneous pollutant breakdown and antibacterial disinfection under visible light.

However, it is critical to note that results obtained from dye or pharmaceutical degradation in water should not be directly extrapolated to food contaminants. Furthermore, degradation percentage alone does not demonstrate detoxification. The toxicity and persistence of degradation products should be considered, particularly for mycotoxins and pesticide residues, where transformation products may retain or even exhibit increased toxicity.

### 5.3 Intelligent Packaging and Nanomaterial‐Based Biosensors

A third convergent application area uses the optical, electronic, and catalytic properties of ZnO and related nanomaterials for rapid, sensitive detection of foodborne pathogens and spoilage indicators, moving food safety monitoring from slow culture‐based methods toward real‐time or near‐real‐time formats. A comprehensive review catalogued advances in nanomaterial‐based biosensors for key foodborne pathogens across the preceding decade, highlighting gains in sensitivity and multiplexing capability enabled by nanoscale transducer materials (Zhang et al. 2020). ZnO‐specific platforms include ZnO‐nanorod‐based electrochemical immunoassays for *E. coli* , in which antibody‐ and redox‐probe‐functionalized, silica‐coated ZnO nanorods enabled detection limits as low as 50 colony‐forming units per milliliter, and ZnO‐nanograss platforms decorated with gold nanospeckles for DNA‐hybridization‐based detection of *Staphylococcus epidermidis* (Bobrinetskiy et al. 2021). While *S. epidermidis* is not a primary foodborne pathogen, this example illustrates the platform technology; future work should prioritize major foodborne pathogens such as *Salmonella*, *Listeria*, and *E. coli* O157:H7.

More recent work has extended these principles to microfluidic, multiplexed, and machine‐learning‐assisted biosensing formats, aiming to shorten detection turnaround from days to hours while retaining or improving sensitivity relative to standard culture‐based enumeration. Nanobiosensor‐integrated intelligent packaging—in which a sensing element is embedded directly within or on the packaging film—has similarly been proposed as a route to continuous, noninvasive monitoring of microbial contamination and spoilage status throughout distribution and retail. Taken together, these three converging application streams—active packaging, photocatalytic decontamination, and nanobiosensing—map onto sequential points along the food value chain, from raw material handling through processing, storage, and point‐of‐sale, as summarized in Figure 2 and Table 2.

**FIGURE 2:**
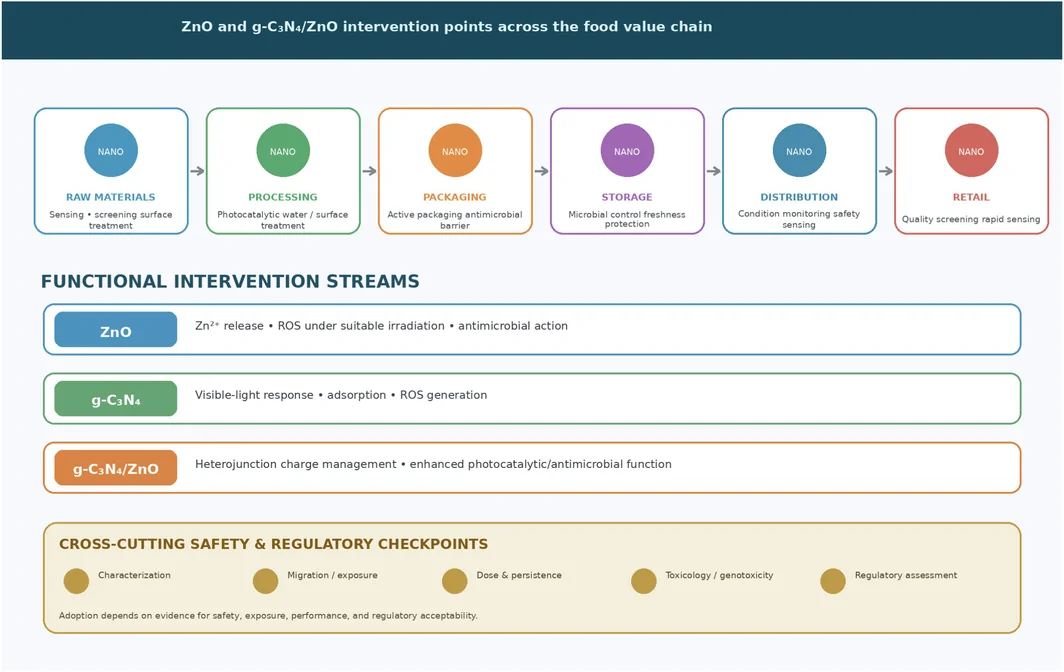
Conceptual framework showing candidate points of intervention for ZnO and g‐C_3_N_4_/ZnO nanomaterials across the food value chain, alongside the cross‐cutting safety and regulatory checkpoints that govern their adoption. [Figure redrawn with clearer representation of demonstrated food applications, sensing, packaging, photocatalytic treatment, and proposed future applications.]

## 6 Toxicological and Regulatory Considerations

Uptake of ZnO and g‐C_3_N_4_/ZnO nanomaterials in the food sector depends on resolving open questions about nanoparticle migration, dose, and long‐term safety. Bulk zinc oxide is classified as Generally Recognized As Safe (GRAS) by regulatory agencies for specific food applications; however, this status does not automatically extend to nanoscale ZnO, which requires separate safety assessment due to altered physicochemical properties and potential for increased cellular uptake. The increased surface‐area‐to‐volume ratio and cellular uptake potential of nanoscale materials mean that toxicological behavior cannot always be extrapolated directly from bulk‐material safety data (Gupta et al. 2024). A 2024 study investigating the toxicological effects of nanomaterials in food packaging highlighted dynamic light scattering and zeta‐potential analysis as key characterization tools for predicting nanoparticle behavior and potential adverse effects in biological systems, and it summarized the regulatory architecture governing food‐contact nanomaterials in both the European Union and the United States (Gupta et al. 2024).

In the European Union, food‐contact nanomaterials fall under the general food‐contact‐materials framework (Regulation (EC) No. 1935/2004) and the plastics‐specific Regulation (EU) No. 10/2011, both of which require case‐by‐case risk assessment for any nanomaterial before approval, with EFSA responsible for evaluating migration behavior, characterization data, and toxicological endpoints (Gupta et al. 2024; Varalda 2024). EFSA's 2021 technical guidance on measurement and risk assessment of nanomaterials in the food and feed chain remains a reference point for migration testing methodology (EFSA 2021), and a 2024 EFSA risk‐assessment review of food‐contact materials more broadly emphasized genotoxicity assessment as a central pillar of the safety evaluation process, given the many thousands of substances—intentionally added and non‐intentionally added—that can migrate from contact materials into food (Varalda 2024). Separately, a 2024 study commissioned by the European Union Observatory for Nanomaterials reported evidence of what it characterized as systematic migration of nanoparticles from food‐contact materials into food, with silver nanoparticles the most extensively studied case, while noting that no general conclusions on toxicity could yet be drawn and identifying substantial further research needs (Food Packaging Forum 2024). However, findings on silver nanoparticle migration should not be directly generalized to ZnO or g‐C_3_N_4_/ZnO without supporting evidence. In the United States, the FDA requires manufacturers to submit safety data for nanomaterials used in food‐contact applications through the Food Contact Notification process under the Food, Drug, and Cosmetic Act (Gupta et al. 2024).

It is important to distinguish the regulatory requirements for different categories of food‐contact materials. Food additives require premarket approval; edible coatings may be regulated as food ingredients or food‐contact materials depending on composition; active packaging that releases substances into food requires migration testing and safety assessment; and non‐release active packaging has different requirements.

These regulatory frameworks continue to evolve. A broader 2025 review of nanomaterial migration and regulatory frameworks in food packaging concluded that existing analytical techniques remain insufficient for reliably tracking the migration of nanoscale organic materials specifically and called for unified analytical methods, improved predictive models, and more adaptable regulatory oversight to keep pace with innovation in nanotechnology‐enabled food‐contact materials. Separately, EFSA's 2025 literature review on micro‐ and nanoplastic release from food‐contact materials—while focused on plastics rather than engineered metal‐oxide nanoparticles—illustrates the broader regulatory attention now directed at particulate release from food‐contact materials during normal use, and identified mechanical stress and fiber shedding as key release mechanisms alongside persistent methodological shortcomings and data gaps across the underlying evidence base (EFSA 2025).

The effects of food composition, pH, temperature, ionic strength, and storage time on nanoparticle migration and Zn^2+^ release are critical considerations. Acidic pH accelerates ZnO dissolution; high ionic strength (common in many foods) can screen electrostatic interactions and alter release profiles; food components such as proteins, amino acids, and organic acids can complex Zn^2+^, reducing free ion concentration while potentially altering bioavailability; and the presence of chelating agents may either enhance or suppress release depending on their binding affinity. Time‐resolved migration studies under realistic food‐contact conditions are needed.

For g‐C_3_N_4_‐containing materials specifically, safety data remain considerably sparser than for ZnO, reflecting the more recent emergence of this class of photocatalyst. Most existing toxicological characterization has focused on environmental remediation contexts (wastewater treatment) rather than direct food‐contact exposure. Data on oral exposure, cellular uptake, and systemic effects relevant to food‐contact exposure are severely limited. This represents a clear evidence gap that we return to in Section 7.

## 7 Challenges and Research Gaps

Several recurring gaps constrain translation of ZnO and g‐C_3_N_4_/ZnO nanomaterials from laboratory demonstration to food‐sector deployment.

First, there is a persistent disconnect between the extensive literature on green‐ and biogenic‐synthesis of ZnO nanoparticles for general antimicrobial or biomedical purposes and the comparatively limited number of studies that test these specific green‐synthesized materials within food‐contact matrices under conditions representative of real food systems, such as complex food matrices, temperature fluctuations during distribution, and co‐contaminating microbiota (Vieira et al. 2026). Research priority: Systematic comparative testing of green/biogenic ZnO nanoparticles against conventionally synthesized counterparts within identical food‐packaging formulations and antimicrobial assay conditions.

Second, photocatalytic degradation efficiencies reported for pure mycotoxin standards in solution do not always translate directly to real food powders or complex matrices, where competing organic material, matrix opacity, and reduced light penetration measurably reduce degradation efficiency, as illustrated by the substantially lower zearalenone degradation observed in real powder samples relative to reference solutions (Huang et al. 2025). Research priority: Systematic testing of photocatalytic performance in real food matrices under conditions approximating commercial processing, with standardized reporting of light source, matrix type, and degradation efficiency.

Third, standardized, harmonized protocols for characterizing nanoparticle migration, size distribution, and surface chemistry in food‐contact contexts remain underdeveloped, a limitation flagged repeatedly in both EFSA‐linked and independent reviews of nanomaterial migration from food‐contact materials, and one that particularly affects organic or metal‐free nanomaterials such as g‐C_3_N_4_, for which analytical detection methods are less mature than for metal or metal‐oxide nanoparticles. Research priority: Development and validation of standardized analytical methods for detecting and quantifying g‐C_3_N_4_ and its derivatives in food matrices.

Fourth, reproducibility and scale‐up of biogenic synthesis routes present practical challenges: microbial culture conditions (temperature, pH, growth phase) influence the protein and metabolite profile available for nanoparticle nucleation and capping, and this biological variability can translate into batch‐to‐batch differences in particle size distribution and polydispersity that are less pronounced in tightly controlled chemical synthesis routes (Patel et al. 2026). Research priority: Development of standardized biogenic synthesis protocols with defined quality‐control criteria for particle size, morphology, and surface chemistry.

Fifth, toxicological and life‐cycle assessment of these materials remains incomplete. Dose–response and chronic‐exposure data specific to food‐relevant exposure routes are limited relative to the volume of antimicrobial‐efficacy data available, and comparative life‐cycle assessments weighing the environmental footprint of nanomaterial‐enabled food packaging against conventional alternatives (including the plastics they may be designed to reduce) are still uncommon in this literature. Research priority: Systematic toxicological characterization encompassing acute and sub‐chronic oral exposure, migration modeling under simulated food‐contact conditions, and genotoxicity screening consistent with EFSA's Note for Guidance framework, extended specifically to g‐C_3_N_4_ and g‐C_3_N_4_/ZnO heterojunctions.

## 8 Future Perspectives

Building on the gaps identified above, we highlight five priorities for future research, clearly distinguishing demonstrated advantages from proposed or potential applications.

First, comparative studies directly benchmarking green/biogenic ZnO nanoparticles—including actinobacterially synthesized variants—against conventionally synthesized ZnO nanoparticles within identical food‐packaging formulations and identical antimicrobial assay conditions would help clarify whether the presumed safety and sustainability advantages of biogenic synthesis translate into measurable differences in food‐relevant performance, rather than remaining largely inferential. Demonstrated: Biogenic synthesis is feasible and produces antimicrobial ZnO. Proposed: Biogenic ZnO offers superior safety and sustainability profiles compared to conventional ZnO—this requires validation.

Second, photocatalytic detoxification studies should increasingly move from mycotoxin standards in solution toward spiked or naturally contaminated real food matrices (grains, feed, juices) under conditions approximating commercial processing (light source, exposure duration, matrix opacity) to generate performance data with more direct relevance to industrial adoption. Demonstrated: Photocatalytic degradation works in model solutions. Proposed: Photocatalytic detoxification will work in real food matrices—this requires demonstration.

Third, systematic toxicological characterization—encompassing acute and sub‐chronic oral exposure, migration modeling under simulated food‐contact conditions, and genotoxicity screening consistent with EFSA's Note for Guidance framework—should be extended specifically to g‐C_3_N_4_ and g‐C_3_N_4_/ZnO heterojunctions, for which such data remain markedly sparser than for ZnO alone. Demonstrated: ZnO has some toxicological data. Proposed: g‐C_3_N_4_/ZnO is safe for food contact—this requires substantial additional data.

Fourth, integration of nanobiosensing with active packaging and photocatalytic decontamination functions into single multifunctional materials—rather than developing each function in isolation—may offer a more commercially compelling value proposition, combining real‐time spoilage/pathogen alerting with active antimicrobial and detoxification capability within one packaging system. Demonstrated: Individual functions (sensing, antimicrobial, photocatalytic) work in isolation. Proposed: Integration of multiple functions into a single material is feasible and beneficial—this requires demonstration.

Fifth, given the divergence between rapidly evolving nanomaterial research and comparatively slower‐moving regulatory frameworks, closer engagement between food‐nanotechnology researchers and regulatory bodies (EFSA, FDA, and their counterparts in other jurisdictions) during the early stages of material development—rather than only at the point of commercialization—may help align research priorities with the specific evidentiary standards regulators will ultimately require, shortening the translational pathway from bench to shelf. Demonstrated: Regulatory frameworks exist and are evolving. Proposed: Early engagement will accelerate translation—this is a reasonable expectation but requires validation.

## 9 Conclusion

Zinc oxide nanoparticles and their heterojunctions with graphitic carbon nitride represent a mechanistically well‐characterized and functionally versatile class of nanomaterials with clear relevance to food safety and preservation. Their antimicrobial activity, rooted in reactive‐oxygen‐species generation, zinc‐ion release, and direct membrane interaction, complements a growing body of evidence for visible‐light‐driven photocatalytic degradation of mycotoxins, pesticide residues, and other organic contaminants, while parallel developments in nanomaterial‐based biosensing offer a route toward faster, more sensitive detection of foodborne pathogens and spoilage. Green and biogenic synthesis routes, including those mediated by soil‐ and plant‐associated *Streptomyces* species, add a further dimension of sustainability to this technology base.

However, while substantial evidence supports the antimicrobial and photocatalytic efficacy of ZnO and g‐C_3_N_4_/ZnO nanomaterials in model systems, translation to real food and food‐contact applications remains limited, with only a small number of studies demonstrating performance under conditions representative of actual food systems. Realizing the full food‐safety potential of these materials will depend on closing persistent gaps in matrix‐representative efficacy testing, standardized migration and toxicological characterization, and regulatory clarity—particularly for the comparatively newer g‐C_3_N_4_‐based systems. Addressing these gaps through the coordinated research priorities outlined above offers a realistic pathway toward safe, standardized, and sustainable adoption of ZnO‐ and g‐C_3_N_4_/ZnO‐based nanotechnologies across the food value chain. However, it must be emphasized that these technologies are not yet ready for standardized food‐chain adoption without sufficient real‐food, toxicological, migration, and regulatory validation.

## Author Contributions

**David Adeiza Zakari:** writing – original draft, conceptualization, methodology, software, data curation, project administration, investigation, validation, writing – review and editing, formal analysis, supervision. **Ibrahim Khalil:** conceptualization, methodology, writing – review and editing. **Kareem Sarafadeen Olateju:** writing – original draft, writing – review and editing, methodology, conceptualization. **Sadullaev Tuymurod Mardullo ugli:** conceptualization, validation, writing – review and editing. **Normirzaev Abdukayum Rakhimberdievich:** conceptualization, methodology, writing – review and editing. **Samandarov Abrorbek:** investigation, conceptualization, writing – review and editing. **Audu Godwin Amoka:** writing – original draft, writing – review and editing, methodology, conceptualization. **Adefila Adebimpe Moyosore:** writing – original draft, conceptualization, methodology, writing – review and editing.

## Conflicts of Interest

The authors declare no conflicts of interest.

## Data Availability

Data sharing is not applicable to this article as no new data were created or analyzed in this review.

## References

[fsn372509-bib-0001] Alameen, A. S. , S. B. Undre , and P. B. Undre . 2024a. “Synthesis, Dispersion, Functionalization, Biological and Antioxidant Activity of Metal Oxide Nanoparticles: Review.” Nano‐Structures & Nano‐Objects 39: 101298. 10.1016/j.nanos.2024.101298.

[fsn372509-bib-0002] Alameen, A. S. , S. B. Undre , and P. B. Undre . 2024b. “Functionalization of ZnO Nanoparticles and Their Antimicrobial Activity: In Vitro.” Nano‐Structures & Nano‐Objects 39: 101314. 10.1016/j.nanos.2024.101314.

[fsn372509-bib-0003] Bobrinetskiy, I. , M. Radovic , F. Rizzotto , et al. 2021. “Advances in Nanomaterials‐Based Electrochemical Biosensors for Foodborne Pathogen Detection.” Nanomaterials 11, no. 10: 2700. 10.3390/nano11102700. PMC8538910 34685143

[fsn372509-bib-0004] European Food Safety Authority (EFSA) . 2021. “Guidance on the Risk Assessment of Nanomaterials in the Food and Feed Chain.” EFSA Journal 19, no. 7: 6768. 10.2903/j.efsa.2021.6768. PMC8331059 34377190

[fsn372509-bib-0005] European Food Safety Authority (EFSA) . 2025. “Literature Review on Micro‐ and Nanoplastic Release From Food Contact Materials During Their Use.” EFSA Supporting Publications 22: 9733. 10.2903/sp.efsa.2025.EN-9733.

[fsn372509-bib-0006] Food Packaging Forum . 2024. “Reviewing Nanomaterials in 2024—Types, Migration, Toxicity.” https://foodpackagingforum.org/news/reviewing‐nanomaterials‐in‐2024‐types‐migration‐toxicity.

[fsn372509-bib-0007] Gökmen, V. , G. G. Gökmen , F. S. Mirsafi , et al. 2024. “Zinc Oxide Nanomaterials: Safeguarding Food Quality and Sustainability.” Comprehensive Reviews in Food Science and Food Safety 23, no. 6: e70051. 10.1111/1541-4337.70051. 39530622

[fsn372509-bib-0008] Gupta, R. K. , P. Guha , and P. P. Srivastav . 2024. “Investigating the Toxicological Effects of Nanomaterials in Food Packaging Associated With Human Health and the Environment.” Journal of Hazardous Materials Letters 5: 100125. 10.1016/j.hazl.2024.100125.

[fsn372509-bib-0009] Hassan, F. , S. N. Backer , I. W. Almanassra , et al. 2024. “Solar‐Matched S‐Scheme ZnO/g‐C_3_N_4_ for Visible Light‐Driven Paracetamol Degradation.” Scientific Reports 14: 12220. 10.1038/s41598-024-60306-0. PMC11585614 38806502

[fsn372509-bib-0010] Huang, Y. , M. Li , and J. Liu . 2025. “Photocatalytic Degradation of Mycotoxins by Heterogeneous Photocatalysts.” Catalysts 15, no. 2: 112. 10.3390/catal15020112.

[fsn372509-bib-0011] Kalaba, M. T. , G. M. El‐Sherbiny , E. A. Ewais , O. M. Darwesh , and S. A. Moghannem . 2024. “Green Synthesis of Zinc Oxide Nanoparticles (ZnO‐NPs) by *Streptomyces baarnensis* and Its Active Metabolite (Ka): A Promising Combination Against Multidrug‐Resistant ESKAPE Pathogens and Cytotoxicity.” BMC Microbiology 24: 254. 10.1186/s12866-024-03392-4. PMC11232237 38982372

[fsn372509-bib-0012] Mirres, A. C. d. M. , I. R. S. Vieira , L. Tessaro , et al. 2024. “Nanocomposite Films of Babassu Coconut Mesocarp and Green ZnO Nanoparticles for Application in Antimicrobial Food Packaging.” Food 13, no. 12: 1895. 10.3390/foods13121895. PMC11203357 38928835

[fsn372509-bib-0013] Patel, P. , M. Al‐zharani , M. Mubarak , et al. 2026. “Biosynthesis of Antibacterial Zinc Oxide Nanoparticles From Endophytic *Streptomyces werraensis* .” Scientific Reports 16: 18021. 10.1038/s41598-026-54975-2. PMC13254299 42270820

[fsn372509-bib-0014] Prathibha, C. P. , S. Mallapur , S. Kota , S. P. Maradur , and S. G. Kumar . 2025. “Facile Preparation of g‐C_3_N_4_/ZnO S‐Scheme Heterojunction for Photocatalysis: Insights From the DFT Studies and Radical Scavenging Experiments.” Chemical Papers 79: 8595–8604. 10.1007/s11696-025-04337-5.

[fsn372509-bib-0015] Varalda, T. 2024. “Risk Assessment of Food Contact Materials.” EFSA Journal 22, no. S1: e221107. 10.2903/j.efsa.2024.e221107. PMC11659736 39712917

[fsn372509-bib-0016] Vieira, I. R. S. , A. C. D. M. Mirres , and C. A. Conte‐Junior . 2026. “Zinc Oxide Nanomaterials: Green Synthesis, Properties, and Potentials in Active Food Packaging, Edible Coatings, and Nanomedicine Applications.” ChemistrySelect 11: e202504228. 10.1002/slct.202504228.

[fsn372509-bib-0017] World Health Organization / Foodborne Disease Burden Epidemiology Reference Group . 2026. “WHO Estimates of the Global, Regional, and National Burden of 42 Foodborne Infectious and Chemical Hazards, 2000–21: An Updated Data Synthesis.” Lancet Global Health 14, no. 8: 1–17. 10.1016/j.langlo.2026.103994 PMC13433341 42235567

[fsn372509-bib-0018] Zhang, H. , X. Wang , J. Liu , et al. 2024. “Alginate Composite Films Incorporated With Zn‐Based Inorganic Antimicrobials for Food Packaging: Effects of Morphology.” Journal of Food Science 89, no. 9: 5734–5747. 10.1111/1750-3841.17272. 39098814

[fsn372509-bib-0019] Zhang, R. , T. Belwal , L. Li , X. Lin , Y. Xu , and Z. Luo . 2020. “Nanomaterial‐Based Biosensors for Sensing Key Foodborne Pathogens: Advances From Recent Decades.” Comprehensive Reviews in Food Science and Food Safety 19, no. 4: 1465–1487. 10.1111/1541-4337.12576 33337098

[fsn372509-bib-0020] Zhou, X.‐Q. , Z. Hayat , D. D. Zhang , et al. 2023. “Zinc Oxide Nanoparticles: Synthesis, Characterization, Modification, and Applications in Food and Agriculture.” PRO 11, no. 4: 1193.

